# Transition from F-75 to ready-to-use therapeutic food in children with severe acute malnutrition, an observational study in Uganda

**DOI:** 10.1186/s12937-017-0276-z

**Published:** 2017-08-30

**Authors:** Betty Lanyero, Hanifa Namusoke, Nicolette Nabukeera-Barungi, Benedikte Grenov, Ezekiel Mupere, Kim Fleischer Michaelsen, Christian Mølgaard, Vibeke Brix Christensen, Henrik Friis, André Briend

**Affiliations:** 1 Mwanamugimu Nutrition Unit, Department of Paediatrics, Mulago National Referral Hospital, P.O. Box 7051, Kampala, Uganda; 2 Department of Nutrition, Exercise and Sports, University of Copenhagen, -1958 Frederiksberg C, DK Denmark; 3 Department of Paediatrics and Child Health, College of Health Sciences, Makerere University, P.O. Box 7072, Kampala, Uganda; 4 Department of Pediatrics and Adolescence, Rigshospitalet, -2100 Copenhagen Ø, DK Denmark; 5 Tampere Centre for Child Health Research, University of Tampere and Tampere University Hospital, Lääkärinkatu 1, 33014 Tampere, Finland

**Keywords:** Severe acute malnutrition, Transition, RUTF, Children, Uganda

## Abstract

**Background:**

World Health Organization now recommends the transition from F-75 to ready-to-use therapeutic foods (RUTF) in the management of severe acute malnutrition (SAM). We described the transition from F-75 to RUTF and identified correlates of failed transition.

**Methods:**

We conducted an observational study among children aged 6–59 months treated for SAM at Mulago hospital, Kampala, Uganda. Therapeutic feeding during transition phase was provided by first offering half of the energy requirements from RUTF and the other half from F-75 and then increasing gradually to RUTF as only energy source. The child was considered to have successfully transitioned to RUTF if child was able to gradually consume up to 135 kcal/kg/day of RUTF in the transition phase on first attempt. Failed transition to RUTF included children who failed the acceptance test or those who had progressively reduced RUTF intake during the subsequent days. Failure also included those who developed profuse diarrhoea or vomiting when RUTF was ingested.

**Results:**

Among 341 of 400 children that reached the transition period, 65% successfully transitioned from F-75 to RUTF on first attempt while 35% failed. The median (IQR) duration of the transition period was 4 (3–8) days. The age of the child, mid-upper arm circumference, weight-for-height z-score and weight at transition negatively predicted failure. Each month increase in age reflected a 4% lower likelihood of failure (OR 0.96 (95% CI 0.93; 0.99). Children with HIV (OR 2.73, 95% CI 1.27; 5.85) and those rated as severely ill by caregiver (OR 1.16, 95% CI: 1.02; 1.32) were more likely to fail. At the beginning of the rehabilitation phase, the majority (95%) of the children eventually accepted RUTF while only 5% completed rehabilitation in hospital on F-100.

**Conclusion:**

Transition from F-75 to RUTF for hospitalized children with SAM by gradual increase of RUTF was possible on first attempt in 65% of cases. Younger children, severely wasted, HIV infected and those with severe illness as rated by the caregiver were more likely to fail to transit from F-75 to RUTF on first attempt.

## Background

Acute malnutrition affects more than 52 million children worldwide [1]. Of these, an estimated 16 million have the severe form of acute malnutrition. Patients with severe acute malnutrition (SAM) are classified into those with and without medical complications depending on the presence of medical illness and/or integrated management of childhood illness (IMCI) danger signs. The treatment of complicated SAM is divided into stabilization and rehabilitation phases, with a period of transition in between. In-patient therapeutic feeding used to involve the use of two milk-based formulae: F-75 (low protein, low energy) in stabilization and F-100 (high protein and high energy) in rehabilitation [2]. Only patients with SAM and medical complications require hospitalization to stabilize while those without medical complications can be managed in the outpatient therapeutic care (OTC) on ready-to-use therapeutic food (RUTF). WHO recommends the use of ready-to-use therapeutic food (RUTF) during the rehabilitation phase [3]. RUTF is a lipid-based therapeutic food whose nutritional composition is similar to F-100. Transition phase feeding refers to the feeding regimen offered to children during the transition phase [4]. During this phase, the therapeutic feed is gradually changed from F-75 to F-100 or RUTF. However, there is no clear guideline on how a gradual transition from stabilization to rehabilitation in the therapeutic feeding can be done [3]. A systematic review done in 2012 on transition approach found no studies that demonstrated the appropriate amounts or composition of feed to be offered after stabilization [5]. WHO suggests a slow and gradual transition phase feeding in which F-100 or ready-to-use therapeutic food (RUTF) is introduced in an equal volume as F-75 for 2–3 days before offering larger volumes intended for catch-up growth in the rehabilitation phase [3].

In 2013, WHO recommended the direct transition from F-75 to RUTF in in-patient settings using RUTF as alternative to previously used F-100 [3]. This would enable the rehabilitation phase of children with SAM to be completed at home while they receive the nutrient-dense RUTF. The success of the community-based management of acute malnutrition (CMAM) approach has been in part due to availability of RUTF that is used for out-patient management of uncomplicated SAM [6].

Based on expert opinion, WHO suggests two approaches to transition from F-75 to RUTF; the first approach involves giving a child RUTF in amounts as prescribed for the transition phase, if the child does not take the prescribed amount of RUTF, then a top-up with F-75 is given. The amount of RUTF is gradually increased over 2–3 days until the child takes the full requirement of RUTF. The second approach involves giving the child the prescribed amount of RUTF for the transition phase. If the child does not take at least half the prescribed amount of RUTF in the first 12 h, the RUTF is stopped and the child is given F-75 again. The same approach is re-tried after another 2 days until the child takes the appropriate amount of RUTF to meet the energy needs [3].

In addition to the limited evidence to the most appropriate approach, limited guidance exists on the estimation of the correct amount of the top-up with F-75 feed without exceeding the energy requirement for this phase of management (100–135 kcal/kg/day). We also do not know the proportion of the children are mostly likely to fail to transit from F-75 to RUTF on first attempt. We describe the results from a clinical study where we transitioned hospitalized children aged 6–59 months with SAM from F-75 to RUTF, using the first approach by first providing half of the energy requirements from RUTF and the other half from F-75 and then increasing gradually to RUTF as only energy source. We then evaluated the correlates of failed transition to RUTF.

## Methods

### Study design

This was a prospective observational study nested within a randomized clinical trial (www.isrctn.com, ISRCTN16454889) investigating the effect of probiotics on diarrhoea in children with SAM.

### Study population

Using the eligibility criteria for the randomized clinical trial, a total of 400 children aged 6–59 months with SAM ((weight-for-height/weight-for-length z-score (WHZ/WLZ) < −3 or mid-upper arm circumference (MUAC) <11.5 cm or bipedal pitting oedema)) [3] were enrolled between March 2014 and July 2015. Children whose caregiver was willing to consent and come back for follow-up were included. Children in shock, severe respiratory distress, admission weight less than 4.0 kg and obvious congenital anomalies were excluded from the study.

### Study setting

The study was conducted in Mwanamugimu Nutrition Unit (MNU) Mulago Hospital, Kampala, Uganda. Mulago hospital serves as the national referral hospital receiving patients from various regions of the country and provides basic health care for the surrounding population. With an 80 bed capacity, MNU admits approximately 100 children monthly, providing both in-patient (ITC) and outpatient therapeutic care (OTC) services for patients with SAM.

### Patient management

All patients in the study received standard treatment in addition to the two probiotic strains or placebo. The standard patient management at MNU followed the integrated management of acute malnutrition guidelines for Uganda adapted from the WHO guidelines [3]. Within the national referral hospital, enrolled patients were screened at acute care unit, then transferred to MNU. A trained nutritionist performed a detailed nutrition assessment while a paediatrician on the study performed evaluation for medical complications. From these two, hospitalization was considered.

In the stabilization phase, patients received F-75 (Nutriset, Malaunay, France) at 100–135 kcal/kg/day and were monitored for improving appetite, resolving medical complications and/or reducing bilateral pedal oedema. When appetite had improved with the child completing more than 80% of therapeutic feeds prescribed for a 24 h period, oedema subsided to grade one or two and medical complications resolving, the patient was considered ready for transition phase.

During transition phase, the therapeutic feed was gradually changed from F-75 to RUTF, Plumpy’nut®, or F-100 both from Nutriset, Malaunay, France. An acceptance test that evaluated whether a child could take RUTF was performed at the start of transition. The acceptance test was conducted in a separate and quiet corner of the ward at 12:00 pm every day, just before feeding time, with the help of the caregiver. The caregiver was informed about the process, asked to wash his/her hands and instructed to give the child small quantities of RUTF directly from the sachet for approximately 30 min while offering safe drinking water. By observation, the study nutritionist considered the test passed if the child completed at least approximately a third of the 92 g sachet. Taking into consideration the recommended energy intake for the transition phase, 100–135 kcal/kg/day, the total intake for the day was calculated and subdivided such that on the first day of transition, 50 kcal/kg was obtained from RUTF and the other 50 kcal/kg from F-75. For the subsequent days, more RUTF than F-75 was prescribed while maintaining the 100–135 kcal/kg/day intake until the child received RUTF alone. A child was considered successfully transitioned on the first attempt from F-75 to RUTF if he/she took 135 kcal/kg/day. The food intake, quantities and tolerance of RUTF were closely monitored by the study nutritionist who also made adjustments in the feeding plan daily.

### Failed transition

In this study, failed transition to RUTF on first attempt was defined as a child who failed the acceptance test despite the improved appetite with F-75 and clinical well state or one who had progressively reduced RUTF intake during the subsequent days or one who developed profuse diarrhoea or vomiting when RUTF was ingested. Those who failed transition to RUTF but were clinically well, received F-100.

During the transition period, the study team monitored the child for appetite, vomiting feeds, profuse diarrhoea and dehydration, re-accumulation of oedema, clinical deterioration in the respiratory rate, pulse rate and temperature or development of negative reactions to the RUTF. In the presence of any of these symptoms and signs, the clinician transferred the child back to F-75 in stabilization phase.

For those that successfully transited to RUTF on the first attempt, they were transferred to complete the rehabilitation phase in the out-patient therapeutic care (OTC). At discharge from hospital, RUTF was prescribed at 200 kcal/kg/day and a follow-up appointment was given. For children who simply rejected RUTF with no negative reactions, another attempt to give RUTF was done after 2–3 days in rehabilitation phase while for those that developed profuse watery diarrhoea and vomiting, no further attempts to RUTF were made. These children completed the rehabilitation phase in hospital on F-100.

### Data collection procedures

A case report form was used to document all data obtained from the caregiver and patient examination findings. At admission, data was collected on age, sex, date of birth, maternal age and education level, previous medical and treatment history of the child and the presenting symptoms. The caregivers were asked to grade the severity of the child’s illness at admission on a visual analogue scale (VAS) ranging from 0 to 10. A full physical examination to include grade of oedema, dehydration status, skin changes and vital signs (respiratory rate, pulse rate and temperature) was performed by a study medical doctor.

Body weight was measured using a digital scale (Seca 813, Hamburg, Germany) to the nearest 100 g. Length/height was measured using an infant length board (Infant/Child Shorr-Board®, Maryland, USA) and mid-upper arm circumference (MUAC) using colour coded tapes (Child 11.5 red/pac-50, UNICEF), both to the nearest 1 mm. Triple measurement for weight, length/height and MUAC were taken and an average obtained. Anthropometric z-scores for weight-for-height (WHZ) and height-for-age (HAZ) were computed using WHO Anthro version 3.2.2. The study paediatrician together with the nutritionists conducted a daily clinical assessment of the study patients including monitoring of vital signs, grade of oedema, appetite, type and amount of feeding regimens given through stabilization, transition and rehabilitation phases.

### Laboratory tests

Blood sampling was performed at admission, discharge and 8 weeks after discharge. At admission, 4 ml of venous blood was collected into heparinized evacuated tubes (Becton Dickinson, USA) to run tests for HIV, blood cell counts, haemoglobin and C-reactive protein (CRP). HIV serological testing was done using rapid tests (Determine HIV-1/2, Abbott Laboratories, USA) and positive test results were further confirmed using HIV 1/2 Stat-Pak Dipstick Assay kit. For children less than 18 months, HIV status was confirmed by an HIV DNA PCR test at Baylor HIV clinic. Samples were analyzed for cell blood counts and haemoglobin at the Uganda cancer institute laboratory. A copy of the results was provided to doctors on the ward to support patient management. A sample of the blood was centrifuged at 1300–2200 g for 10 min, stored at −80 °C and shipped on dry ice to the laboratory at Department of Nutrition Exercise and Sports, University of Copenhagen, where C-reactive protein was analyzed using Pentra 400, (Horiba ABX, France).

### Data analysis

All data was entered into Epidata version 3.1 and analyzed using Stata version 12. Descriptive statistics using means, medians and proportions was used to present the socio-demographic characteristics, maternal characteristics and laboratory data. To determine the strength of association for the predictors of failed transition from F-75 to RUTF, a series of logistic regression models were fitted. Duration of stabilization phase, HIV infection, severity of illness of the child at admission in addition to age and sex were evaluated as potential confounders. Each of these factors did not change the unadjusted odds ratio by more than 10% hence not included in the final regression model. The independent variables were adjusted for age and sex. *P*-values below 0.05 were statistically significant.

## Results

### Socio-demographic characteristics

The mean (±SD) age of the children that reached transition phase was 17.1 (±8.7) months and 42% were females (Tabl 1). Their mean WHZ and HAZ were −2.5 (±1.5) and −3.1 (±1.4), respectively. At admission, 66% of children presented with oedema. The mean maternal age was 25 (±5.8) years, with 185 (58%) of the mothers having attained primary level education or lower (data not presented).

**Table 1:** Characteristics of 341 severe acute malnutrition children reaching transition^a^

Child characteristics		
Age^a^, months	341	17.1 ± 8.7
Female sex, n (%)	341	143 (42%)
How sick at admission, VAS^b^	340	6.0 ± 1.8
Weight-for-height z	339	−2.5 ± 1.5
Weight for height z score		
≥ −2		125 (37%)
< −2 and > − 3		65 (19%)
< −3 and > −4		90 (27%)
< −4 and > −5		44 (13%)
≤ −5		14 (4%)
Height-for-age z	339	−3.1 ± 1.4
Mid-upper arm circumference, cm	341	11.6 ± 1.5
HIV status, n (%)	323	
Negative		229 (70.9%)
Positive		32 (10%)
Exposed, negative		62 (19%)
Symptoms on admission, n (%)		
Cough	340	225 (66%)
Diarrhea	340	194 (57%)
Fever	340	180 (53%)
Clinical data on admission, n (%)		
Oedema	341	
Grade 1		22 (7%)
Grade 2		64 (19%)
Grade 3		139 (41%)
Flaky paint dermatosis	341	22 (6%)
Laboratory data on admission		
Serum C-reactive protein, mg/dl		
> 10	303	183 (60%)
Hemoglobin	259	8.8 ± 2.1
Clinical data at transition		
Oedema, n (%)	341	
Grade 1		58 (17%)
Grade 2		5 (2%)
Weight, kg	294	6.7 ± 1.6
No. of days with diarrhoea in stabilization	339	4.1 ± 3.9

^a^Data are number of children with data, and mean ± standard deviation or number (%)

^b^ visual analogue scale

### Transition from F-75 to RUTF

**Fig. 1:**
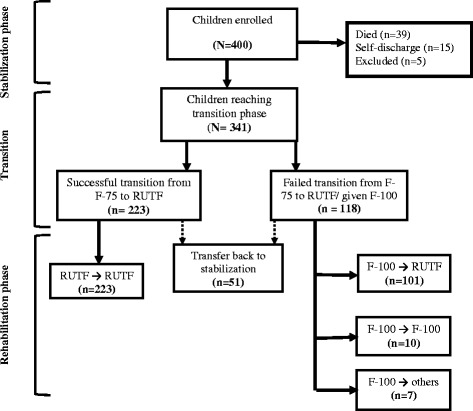
Proportion of children with severe acute malnutrition that failed or succeeded transition from F-75 to RUTF on first attempt. 118 children failed transition to RUTF. Those who failed were children who failed the acceptance test or who had progressively reduced RUTF intake during the subsequent days or developed negative reactions such as profuse diarrhoea or vomiting when RUTF was ingested. Those who failed were given F-100. At beginning of rehabilitation phase, another attempt to RUTF was done for those who had failed transition to RUTF. Of the 118 children, 101 changed feed from F-100 to RUTF, while 10 children remained on F-100 and 7 received other feeds such as porridge. Among the 341 children that were transition, 51 developed signs and symptoms of clinical deterioration while in either transition or rehabilitation phase and were transferred back stabilisation

Of 400 children enrolled, 341 (85%) reached transition phase (Fig. 1). Of these, 223 (65%) succeeded in transition from F-75 to RUTF on the first attempt while 118 (35%) failed (Fig. 1). It took on average 1.4 (±1.4) days to gradually change the feed from RUTF with top up of F-75 to RUTF alone during the transition period. Twelve percent (27/223) of children transitioned directly from F-75 to RUTF without the need for top-up with F-75.

Overall, the median (IQR) duration for the entire transition period was 4 (3–8) days, which included time to transit from F-75 to RUTF or F-100 and resolution of medical complications. However, this duration was longer for those that failed transition to RUTF on the first attempt, [4 (3–6) days vs 7.5 (4 .0–11) days, *p* < 0.01]. There was no difference in number of days in stabilization phase among those succeeding compared to those failing to transition to RUTF (10.5 vs. 9.6 days, *p* = 0.15).

The children that were successfully transitioned to RUTF at the first attempt were then transferred to complete rehabilitation in OTC. On the second attempt for acceptance of RUTF during rehabilitation phase, 101 (86%) of the 118 that received F-100 now accepted the prescribed RUTF and were transferred to OTC to complete rehabilitation. The remaining 14% (17/118) children did not accept RUTF at all. The children that did not accept RUTF at all continued with the rehabilitation phase to attain catch-up growth in hospital while receiving F-100 and allowed home after full recovery on locally available foods (Fig. 1).

### Correlates of failed transition

The age of a child was negatively associated with failed transition from F-75 to RUTF on the first attempt. An odds ratio of 0.96 (95% confidence interval (CI) 0.93; 0.99) for age reflects a 4% lower likelihood of failure to transition from F-75 to RUTF for each month increase in age (Table 2). A WHZ lower than −3 was associated with higher odds of failure (Table 2). Increasing MUAC (OR 0.79, 95% CI 0.68; 0.94) and weight at time of transition (OR 0.68, 95% CI: 0.53; 0.86) were associated with a lower risk of failure. We found a 32% reduction in the risk of failure for every kilogram increase in the weight at the time of transition. The HIV positive children were more likely to fail transition to RUTF (OR 2.73, 95% CI: 1.27; 5.85). The severity of illness of the child as rated by the caregiver at admission using visual analogue scale was found to be associated with failure (OR 1.16, 95% CI: 1.02; 1.32). Neither symptoms of cough, diarrhoea, fever nor presence of oedema whether at admission or at the time of transition were associated with failure (Table 2).

**Table 2:** Correlates of failed transition from F-75 to RUTF among 341 children treated for severe acute malnutrition^a^

	Unadjusted			Age and sex adjusted		
	OR	95% CI	*p*	OR	95% CI	*P*
**Child characteristics**						
Age, months	0.96	0.93;0.99	0.02	0.96	0.93;0.99	0.01
Female sex, n (%)	0.83	0.53;1.31	0.42	0.80	0.51;1.28	0.37
How sick, VAS^b^	1.14	1.01;1.29	0.03	1.16	1.02;1.32	0.02
HIV status, n (%)						
Exposed	1.03	0.56;1.86	0.91	0.96	0.52;1.79	0.92
Positive	2.58	1.22;5.48	0.01	2.73	1.27;5.85	0.02
Weight-for-height z	0.77	0.68;0.91	0.001	0.82	0.69; 0.95	0.01
Weight-for-height z						
≥ −2	ref					
< −2 and > − 3	1.37	0.71; 2.68	0.34	1.31	0.67; 2.57	0.42
< −3 and > −4	2.42	1.35; 4.34	0.003	2.13	1.17; 3.88	0.01
< −4 and > −5	2.10	1.01; 4.33	0.04	1.81	0.86; 3.81	0.12
≤ −5	3.03	0.98; 9.32	0.05	2.52	0.81; 7.94	0.11
Height-for-age z	0.91	0.78;1.06	0.24	0.86	0.72;1.02	0.07
MUAC, cm	0.77	0.66;0.89	0.001	0.79	0.68;0.94	0.01
**Symptoms on admission (%)**						
Cough	1.05	0.65;1.69	0.83	1.04	0.64;1.67	0.89
Diarrhea	1.03	0.66;1.62	0.87	1.00	0.62;1.59	0.99
Fever	1.27	0.81;1.98	0.30	1.27	0.80;2.01	0.31
**Clinical signs on admission (%)**						
Oedema						
Grade 1	1.41	0.56;3.54	0.46	1.55	0.61;3.93	0.35
Grade 2	1.08	0.58;2.03	0.79	1.20	0.63;2.28	0.57
Grade 3	0.68	0.41;1.16	0.16	0.80	0.46;1.38	0.42
Flaky paint dermatosis	1.08	0.44;2.66	0.86	1.12	0.45;2.78	0.80
**Laboratory data on admission**						
C-reactive protein, mg/l						
> 10	0.97	0.60;1.57	0.92	0.98	0.61;1.60	0.96
Hemoglobin, g/dl	0.96	0.85;1.08	0.52	0.96	0.84;1.08	0.52
Weight at transition, kg	0.73	0.61;0.86	<0.001	0.68	0.53;0.86	0.002
No. of days with diarrhoea during stabilization	1.01	0.95;1.07	0.80	0.99	0.94;1.06	0.97
No. of days of stabilization phase	1.02	0.98;1.07	0.31	1.02	0.97;1.06	0.36

^a^Data are odds ratio (OR), 95% confidence interval (CI) and *p*-values

^b^visual analogue scale

### Transfer back to stabilization

Of the 341 children that reached transition phase, 51 (15%) were transferred back to stabilization phase. The clinical characteristics of the children on the day of transfer back to stabilization are presented in Table 3. Nearly half (47%) of those transferred back to stabilization had an increased respiratory rate and 18% were diagnosed to have severe pneumonia (Table 3). Age, lower WHZ score, MUAC and weight at transition were negative predictors of transfer back to stabilization (Table 4).

**Table 3:** Clinical characteristics at the time of transfer back to stabilization ^1^

Clinical characteristics ^*^	*N* = 51
Weight, kg	6.15 ± 1.32
Temperature, °C	36.9 ± 0.83
Pulse rate ^2^, b/min	128 ± 15
Oxygen saturation^3^, (%)	92.3 ± 5.8
Visual analogue scale, VAS	3.0 ± 3.1
Respiratory rate	
Normal	27 (53%)
Increased	24 (47%)
Chest in-drawing	
No	41 (80%)
Yes	10 (20%)
Breath sounds on auscultation	
Normal	41 (80%)
Crepitations	6 (12%)
Diminished	1 (2%)
Pneumonia	
None	37 (72%)
Pneumonia	5 (10%)
Severe pneumonia	9 (18%)
Oedema	
None	45 (88%)
Grade 1	3 (6%)
Grade 2	2 (4%)
Grade 3	1 (2%)
ReSoMal prescribed^4^	
None	33 (65%)
Plan A	10 (20%)
Plan B	8 (15%)
Appetite	
None	5 (10%)
Poor	15 (29%)
Good	31 (61%)

^1^Data presented are mean (SD) or n (%)

^2^N = 13

^3^N = 7

^4^ReSoMal Plan A; 30-50mls of ReSoMal per loose stool for children with no dehydration, ReSoMal plan B; 5mls/kg for first 30 min followed by 5-10mls/kg of ReSoMal for 6–8 h given for some or severe dehydration

^*^A child could have one or more of these characteristics

**Table 4:** Correlates of transfer back to stabilization among 341 children treated with severe acute malnutrition ^a^

	Unadjusted			Age and sex adjusted		
	OR	95% CI	*p*	OR	95% CI	*p*
**Child characteristics**						
Age, months	0.97	0.93; 0.99	0.04	0.96	0.93; 0.99	0.04
Female sex, n (%)	0.78	0.47; 1.29	0.33	0.76	0.45; 1.26	0.29
Severity of illness, VAS^b^	0.83	0.72; 0.94	0.01	0.83	0.73; 0.95	0.01
HIV status, n (%)						
Exposed	0.74	0.37; 1.49	0.40	0.72	0.36; 1.47	0.37
Positive	1.62	0.73; 3.56	0.23	1.68	0.75; 3.72	0.20
Weight-for-height z	0.72	0.61; 0.86	<0.001	0.75	0.62; 0.89	0.002
Height-for-age z	0.95	0.80; 1.13	0.59	0.91	0.76; 1.09	0.33
MUAC, cm	0.77	0.64; 0.91	0.003	0.79	0.66; 0.95	0.01
**Symptoms on admission (%)**						
Cough	1.16	0.68; 1.97	0.58	1.14	0.67; 1.95	0.62
Diarrhea	0.92	0.56; 1.51	0.73	0.87	0.53; 1.45	0.61
Fever	0.94	0.57; 1.54	0.81	0.94	0.57; 1.55	0.81
**Clinical signs on admission (%)**						
Oedema						
Grade 1	1.12	0.42; 3.00	0.81	1.22	0.45; 3.28	0.69
Grade 2	0.80	0.40; 1.61	0.54	0.86	0.43; 1.75	0.68
Grade 3	0.55	0.31; 0.99	0.05	0.63	0.34; 1.15	0.13
Flaky paint dermatosis	0.91	0.32; 2.54	0.855	0.94	0.33; 2.66	0.91
**Laboratory data on admission**						
C-reactive protein, mg/l						
> 10	0.87	0.51; 1.47	0.61	0.88	0.52; 1.49	0.64
Hemoglobin, g/dl	1.12	0.98; 1.29	0.09	1.13	0.99; 1.29	0.06
**Clinical data at transition**						
Edema at time of transition						
Grade 1	0.53	0.24; 1.12	0.10	0.53	0.23; 1.16	0.11
Grade 2	1.91	0.31; 11.6	0.48	3.28	0.47; 22.45	0.23
Weight at transition, kg	0.72	0.58; 0.87	0.001	0.64	0.48; 0.84	0.001
Diarrhoea during stabilization	1.02	0.96; 1.09	0.39	1.02	0.96; 1.08	0.54
Duration of stabilization phase	0.97	0.92; 1.02	0.27	0.96	0.92; 1..02	0.24

^a^Data are odds ratio (OR), 95% confidence interval (CI) and *p*-values

^b^ visual analogue scale

## Discussion

WHO recommends a gradual process of transition from F-75 to RUTF or F-100 [3]. However, the approach to transition process especially to RUTF is lacking in evidence. This study describes a method of gradual transition from F-75 to RUTF with a structured step up of amount of RUTF to be given during the transition phase. We found that 65% of children with SAM transitioned from F-75 to RUTF on first attempt after the stabilization phase while 35% failed. This provides good evidence to support the recommendation by WHO. A recent study conducted in Malawi [7] has further showed that the introduction of RUTF during transition phase has no effect on the stool pH or duration of hospital stay compared to F-100. Furthermore, we note that the majority (95%) of the children in this study later accepted RUTF in the rehabilitation phase with only 5% not taking RUTF at all. That the majority of the children eventually accept RUTF as they begun the rehabilitation phase is relevant information because it implies that many more children can then be managed on an outpatient basis following stabilization of their medical complications. Since endorsement of the CMAM approach by the UN agencies [8], the use of RUTF in the management of uncomplicated SAM has saved millions of lives of children through improved coverage of nutrition programmes, reduced mortality rates, timely treatment of SAM and reduced costs of hospital care [6, 9–11]. This study provides evidence that supports the direct transition from F-75 to RUTF and subsequently these children are managed in the out-patient therapeutic programmes. It is also important to note that not all children were able to transition successfully from F-75 to RUTF at the same speed.

We found that the children that transitioned successfully from F-75 to RUTF on the first attempt, the gradual increase in RUTF till they were on RUTF alone took between 1 and 2.8 days. This is consistent with the current recommendation of 2–3 days [3] hence further strengthens the current recommendation. In this study, the entire transition period lasted 3–8 days among all children that reached transition. This finding is in line with the WHO 1999 guidelines [2] showing transition period to last for 3–7 days. However this transition period was longer for those that failed to transit smoothly to RUTF.

### Predictors of failed transition

Increasing age was associated with a lower risk of failure. Younger children perhaps could have some difficulty with the consistency of RUTF in its current semi-solid state. Coupled with the physiological changes of reduced muscle mass to include the muscles of mastication, intake of RUTF may be somewhat affected compared to F-75 or F-100 that is in liquid form, easier to drink and swallow. It is also probable that these children with malnutrition also have delayed introduction of complementary feeds and commonly the first complementary feeds are fluid in consistency. Studies in an urban district of Nairobi [12] and a rural district in Uganda [13] showed higher probability of receiving liquid to a semisolid or solid complementary food among children in the developing countries. Another study conducted in the western rural district in Uganda [14] found 19% of children in the community aged 6–8 months were still exclusively breastfeed instead of receiving complementary feeds. A review on the global perspective of complementary feeding [15] revealed that a cereal based porridge often with plenty of water was the main complementary food given in many developing countries. A study conducted in Bangladesh [16], 78% of malnourished pregnant and lactating women found the RUTF unacceptable. They further suggested making changes to taste, smell and consistency to make it more liquid [16].

Furthermore, we found that wasting as measured by decreasing WHZ and MUAC were associated with a higher risk of failure. The odds of failure were higher with decreasing initial WHZ. Children with WHZ less than −5 SD were 3 times more likely to fail to transit to RUTF on the first attempt. Probably the most wasted children having been through longer periods of starvation in addition to functional and structural changes in the body organs due to reductive adaptation will similarly require longer period to re-adapt when therapeutic feeding is initiated.

HIV infection and severity of illness as reported by the caregiver were positively associated with higher risk of failure to transit to RUTF. Contrary to this finding, studies conducted in Tanzania [17] and Malawi [18], RUTF has been used successfully among the HIV infected children without medical complications during the home-based therapy of SAM with good results in weight gain. However the study in Malawi [18] showed only 56% vs 84% of children with full recovery in WHZ among the HIV-infected children compared to the HIV-negative children respectively. This was attributed to development of cough, fever and diarrhoea. A study by Zeitz et al. [19] documented lactase deficiency in nearly 50% in HIV patients hence increasing the risk of malabsorption and diarrhoea. This points to probable differences in the metabolic alterations in HIV-infected compared to the HIV-negative children that could equally influence transition to RUTF. Use of RUTF in HIV-infected children merits further evaluations to improve nutrition outcomes.

We found 15% of the children in this study were transferred back to stabilization phase after successful transition due to clinical deterioration with symptoms and signs of increased respiratory rate, pneumonia and diarrhoea with dehydration. Systematic reviews [5, 20] indicate that clinical manifestations of respiratory distress, cardiac failure or lethargy while in transition phase may often be misinterpreted for sepsis or pneumonia yet they are the manifestations of refeeding syndrome. A review of refeeding syndrome [21] highlighted that the severely wasted individuals with prolonged fasting or low energy diet intake were at a higher risk of refeeding syndrome. Refeeding syndrome commonly presents with symptoms and signs of hypophosphatemia and hypomagnesaemia such as respiratory distress, weakness, cardiac failure, nausea and diarrhoea [21]. However recent studies conducted in Uganda [22, 23] showed increasing levels of plasma phosphate in the transition phase and at discharge among children managed for SAM. This follows the WHO recommendations that improved phosphate content in the therapeutic feeds hence refeeding syndrome is unlikely.

We did not find any correlation between failed transition and oedema either at admission or at the time of transition. This supports the recommendation for the use of RUTF when oedema is grade II or I as is done in the outpatient therapeutic programmes [3].

Other factors we would have liked to measure as potential effect modifiers are the timing of introduction of complementary feeds, the type of complementary feeds introduced and previous exposure to RUTF. The previous exposure to RUTF in the children would probably ease the intake of RUTF since this is a taste and consistency the child has been exposed to or if the child did not like it previously, he would reject it when it is given this time. During the selection of study participants, we excluded the children with cerebral palsy who are known to have feeding difficulties. This group of children would have modified the effect on the estimate as they are more likely to fail.

### Strengths and limitations

This is among the first studies that describes the transition feeding of hospitalized children with SAM following the recent WHO recommendations. The study was conducted in a controlled environment during a clinical trial. This facilitated close monitoring of the feeding in children both during day and night.

Amongst the study limitations, firstly, the study selection criteria did not include the severely ill children and those with disabilities hence may affect the external validity of the findings. Secondly, the process of determining whether a child was ready for transition phase as this process is relatively subjective and dependent on the return of appetite. There is no literature that demonstrates sensitivity or specificity of the return of appetite in correctly identifying those ready for transition. It is also not certain whether the refusal of RUTF was because of the new taste to the child rather than a different consistency from the F-75 previously taken during stabilization. However the process and monitoring of transition phase was conducted by well trained and experienced nutritionists. Lastly, we were not able to carry out blood tests for serum electrolytes such as phosphate, magnesium, and potassium during transition phase. Re-feeding syndrome is associated with hypophosphatemia, hypomagnesemia and hyperkalemia. Therefore we cannot say for sure that signs of respiratory distress, diarrhoea observed in the children who were transferred back to stabilization were a result of refeeding syndrome.

## Conclusion

Transition from F-75 to RUTF during the transition phase for hospitalized children with SAM was possible on first attempt in the majority of children with SAM. The method of first providing half the energy requirements using RUTF and the remaining half by F-75 then gradually increasing to RUTF as the only source of energy during transition provides guidance for practitioners managing hospitalized children with SAM.Younger children, severely wasted, HIV infected and those reported to be severely ill by the caregiver were more likely to fail transition to RUTF on first attempt. The transition process from F-75 to RUTF warrants further research to evaluate the most appropriate method of transition with minimal failures and to advance in more objective and field appropriate methods to determine readiness to transit or stabilization of metabolic alterations in children with SAM.

## Abbreviations

CMAM: Community Management of Acute Malnutrition
HIV: Human Immunodeficiency Virus
ITC: In-patient therapeutic care
MNU: Mwanamugimu Nutrition Unit
MUAC: Mid-upper arm circumference
OTC: Out-patient therapeutic care
RUTF: Ready-to-use therapeutic food
SAM: Severe acute malnutrition
VAS: Visual Analogue scale
WHO: World Health Organization
